# Photobiocatalytic CO_2_ reduction into CO by organic nanorod-carbon monoxide dehydrogenase assemblies: surfactant matters†

**DOI:** 10.1039/d4sc03154g

**Published:** 2024-09-18

**Authors:** Mariia V. Pavliuk, Maximilian Böhm, Janna Wilhelmsen, Steffen Hardt, Henrik Land, Haining Tian

**Affiliations:** a Department of Chemistry – Ångström Laboratory, Physical Chemistry, Uppsala University Uppsala Sweden haining.tian@kemi.uu.se; b Department of Chemistry – Ångström Laboratory, Molecular Biomimetics, Uppsala University Uppsala Sweden; c Leiden Institute of Chemistry, Energy and Sustainability – Catalysis and Surface Chemistry, Leiden University Einsteinweg 55 2333 CC Leiden the Netherlands

## Abstract

Photobiocatalytic CO_2_ reduction represents an attractive approach for conversion of solar light and abundant resources to value-added chemicals. However, the design of suitable systems requires a detailed understanding of the interaction between the artificial photosensitizer and biocatalyst interface. In this work, we investigate the effect of surfactant charge utilized in the preparation of a phenoxazine-based organic molecule nanorod photosensitizer on the interaction with the carbon monoxide dehydrogenase II from *Carboxydothermus hydrogenoformans* within biohybrid assemblies for sacrificially driven photobiocatalytic CO_2_ reduction into CO. Electrophoretic mobility shift assay in conjunction with cryogenic electron microscopy (Cryo-EM) and detailed physicochemical characterization are conducted to understand the interaction at the biohybrid interface in order to suggest a strategy for future functionalization of nanoparticles that fulfills the needs of the biocatalyst for green fuel production.

## Abstract 2

Organic nanorods have been prepared to be assembled with carbon monoxide dehydrogenase for photobiocatalytic CO_2_ reduction. The surfactants used to synthesize organic nanorods have shown significant effect on the performance of biohybrid assemblies.

## Graphical abstract

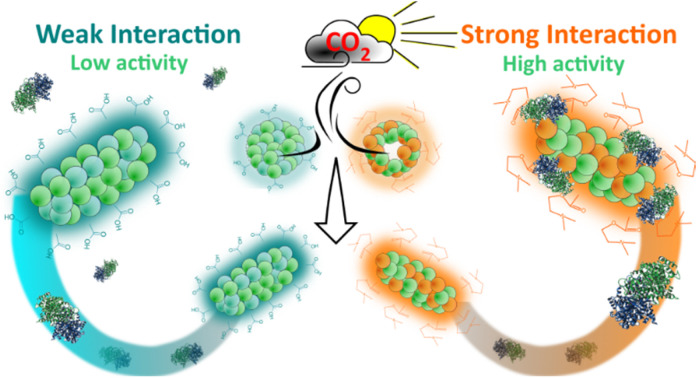

## Introduction

The significance of CO_2_ fixation is self-evident, as it can close the loop of the anthropogenic carbon cycle, additionally allowing production of high-value-added chemicals, *e.g.* renewable fuels.^1^ Photocatalytic CO_2_ fixation is one of the strategies where sunlight is used as the sole energy input to drive CO_2_ reduction into valuable fuels or chemicals. To implement this photocatalytic approach, an efficient catalyst is required to have good selectivity of product formation from the CO_2_ reduction reaction, while a photosensitizer should be used to harvest solar light and provide photogenerated electrons. As compared to inorganic materials or molecular catalysts,^2–6^ nature's enzymes such as nickel containing carbon monoxide dehydrogenase (CODH) is a biocatalyst that catalyses the interconversion between CO_2_ and CO with excellent selectivity and efficiency, thereby motivating studies of its biotechnological application.^6–8^ The fusion of photosensitizers and biocatalysts to facilitate photocatalysis, called photobiocatalysis (also known as semi-artificial photosynthesis or bio-hybrid photocatalysis),^9–12^ has recently gained increasing attention, as it provides unexplored opportunities to achieve synergistic effectiveness for the production of desired biofuels.^13–17^

**Fig. 1:**
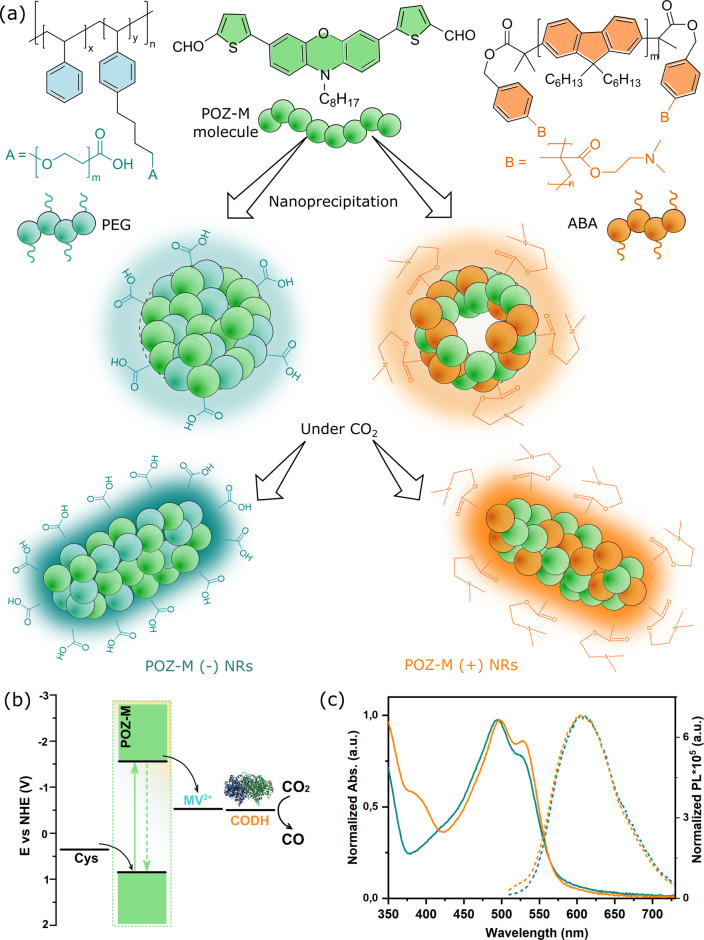
(a) Chemical structures of POZ-M and surfactants (PS-PEG-COOH and ABA) used for the preparation of NRs *via* a modified nanoprecipitation approach. (b) Energy level alignment of POZ-M NRs, cysteine (Cys), redox shuttle MV^2+^, CODH enzyme and CO_2_ reduction potential at pH 5.7. (c) Normalized UV-vis absorption (solid lines) and steady-state photoluminescence (dashed lines) spectra of POZ-M(+) NRs (orange lines) and POZ-M(−) NRs (blue lines) in water.

To photochemically drive CODH for CO_2_ reduction into CO, various photosensitizers have been used,^18^*e.g.* CdS and CdS/CdSe nanorods,^19,20^ Ru complexes on metal oxide nanoparticles (NPs), Ag nanoclusters tethered to TiO_2_ NPs or dye-sensitized NiO.^21–24^ The previous photosensitizers have either toxic/precious metals, poor stability or limited light harvesting ability. To address this, in the current work we adapted a biocompatible small organic molecule nanorod photosensitizer based on a phenoxazine chromophore (named POZ-M,^25^ see Fig. 1) with strong absorption up to 650 nm to efficiently drive CODH II from *Carboxydothermus hydrogenoformans* for sacrificially driven photocatalytic CO_2_ reduction into CO. Recently it was emphasized that interaction at the abiotic–biotic interface plays a crucial role in the rational design of future biohybrid systems.^17,26^ This motivated us to investigate such interactions in detail within the POZ-M:CODH assembly. For this, POZ-M organic NPs have been modified with differently charged surfactants to promote electrostatic interaction with the biological counterpart for CO_2_ reduction.

## Results and discussion

The detailed method of the synthesis of small organic molecule POZ-M NPs and their characterization can be found in the ESI.†Fig. 1 depicts the chemical structures of POZ-M and amphiphilic polymer surfactants (PEG-PS-COOH and ABA) used in the preparation of negatively charged POZ-M(−) NPs and positively charged POZ-M(+) NPs, respectively. In the POZ-M molecule two thiophene π units are covalently linked to the phenoxazine core and two aldehyde units act as electron-withdrawing units to increase intramolecular charge separation. Both POZ-M(−) and POZ-M(+) NPs as prepared formed a stable dispersion with an average particle size of 38 and 43 nm respectively, as determined by dynamic light scattering (DLS, Fig. S1†). Negative surface charge of POZ-M(−) NPs (*ξ* = −24 mV) arose from the carboxyl groups of the PEG-PS-COOH surfactant (Fig. 1a and S2†), while tertiary amine groups of the ABA surfactant susceptible to interaction with CO_2_ were responsible for the positive surface charge of POZ-M(+) NPs (*ξ* = +26 mV).

Cryogenic transmission electron microscopy (Cryo-EM) was conducted to investigate the morphologies of organic nanoparticles used in this study. According to Cryo-EM both NPs looked amorphous without clear signs of a layered or crystalline structure. Specifically, the freshly prepared POZ-M(−) NPs had a spherical porous morphology, while POZ-M(+) NPs had an elongated hollow morphology (Fig. S3†). However, once CO_2_ has been purged to the NP solutions, Cryo-EM studies revealed that both POZ-M(−) NPs and POZ(+) NPs underwent morphology changes from spherical and elongated hollow morphology respectively (Fig. S3†) into nanorod (further denoted as NR) architectures with a more pronounced crystalline structure (Fig. 1a and 3a, b). Morphology changes most likely originate from the seaweed-type orientation of the hydrophobic chains of light-harvesting POZ-M molecules. As a similar morphology change was observed for both positively and negatively charged NPs in the presence of Ar as a carrier gas, it was suggested that NRs are the thermodynamic product of POZ-M NPs (Fig. S4†). POZ-M(−) NRs as well as POZ-M(+) NRs are offering an accessible surface for interaction with the enzyme.

Corresponding energy levels of all materials used for CO_2_ reduction were determined using differential pulse voltammetry,^25^ and are presented in Fig. 1b. As broad light harvesting is an important parameter for efficient solar fuel formation, we have recorded UV-vis absorption spectra of the POZ-M molecule (Fig. S5†) and POZ-M NRs with different charged surfactants (Fig. 1c). As seen from non-normalized absorption spectra (Fig. S6†) both POZ-M(−) and POZ-M(+) NRs had similar absorption profiles extending up to 650 nm. Though minimal differences were observed in the absorption profile of POZ-M NRs with different surfactants, their photoluminescence quantum yields (PLQYs) varied to a larger extent. The PLQY of positively charged POZ-M(+) NRs was two times higher than the PLQY of POZ-M(−) NRs (PLQY_POZ-M(+)_ = 1.5% and PLQY_POZ-M(−)_ = 0.7%) which could be attributed to different degrees of molecular packing and dissimilar chemical environments inside the NRs caused by different amphiphilic polymeric surfactants. In view of the fact that the emission of the ABA surfactant overlaps with the absorption of POZ-M, energy transfer is indeed a possible pathway, which results in suppressed emission at the donor side (∼450 nm), and enhanced emission intensity at the acceptor side (POZ-M, 630 nm, see Fig. S7†), and hence causes the difference in PLQY. The stronger photoluminescence also indicates that the excited molecules in POZ-M(+) have a long-lived excited state as compared to that in POZ-M(−), which generally should be beneficial for photocatalysis, if we consider that the light generated excitons need an adequate lifetime to dissociate well at the interface between the photosensitizer and redox mediator/catalyst.^27–29^

In all cases biohybrid assemblies of organic NRs and CODH were prepared as follows. CODH was injected into the solution of either POZ-M(−) NRs or POZ(+) NRs kept in a sealed vial under a CO_2_ atmosphere inside a glovebox due to the O_2_-sensitivity of the enzyme. Surface *ξ*-potential studies revealed that under photocatalytic conditions (pH 5.7, under a CO_2_ atmosphere), the net surface charge of POZ-M(−) NRs and POZ(+) NRs was +59 mV and −10 mV, while the net surface charge of the CODH enzyme was −30 mV (theoretical isoelectric point, pI = 6.58). Anaerobic incubation with CODH resulted in the net surface charge change to 37 mV for POZ-M(+) NRs, and to – 2 mV for POZ-M(−) NRs, respectively (Fig. S8†).

**Fig. 2:**
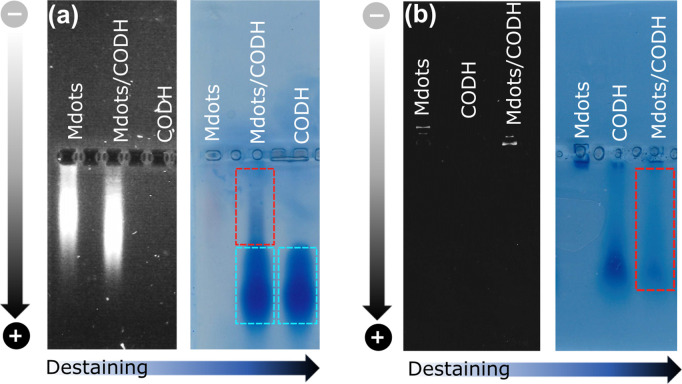
Electrophoretic mobility shift studies for (a) POZ-M(−) and (b) POZ-M(+) NRs in the presence of the CODH enzyme after applied potential (black and white images) and after staining/destaining (light blue image).

The interaction between POZ-M NRs and CODH was further probed by an electrophoretic mobility shift assay as shown in Fig. 2. Negatively charged POZ-M(−) NRs in isolation propagated slower than those NRs with the incubated CODH enzyme. After staining the gel with Coomassie brilliant blue and successive destaining, it reveals that the POZ-M(−) NRs with CODH has a delayed band that arose from the interaction between the two macromolecules. Notably, we have found out that a significant fraction of the sample propagated similar to the bare enzyme which could be attributed to the enzyme that was not adsorbed on the surface of POZ-M(−) NRs most likely due to electrostatic repulsion. At the same time positively charged POZ-M(+) NRs without an enzyme did not propagate through the gel. After the Coomassie staining procedure we observed a far fainter band corresponding to the free enzyme. This suggests that the enzyme adsorbed on the surface of positively charged POZ-M(+) NRs remained in the well due to strong electrostatic interaction with POZ-M(+) NRs. We hypothesize that POZ-M NRs are most likely attracted to the surface of the CODH enzyme *via* a combination of electrostatic interaction, van der Waals interaction and hydrophobic effects.

**Fig. 3:**
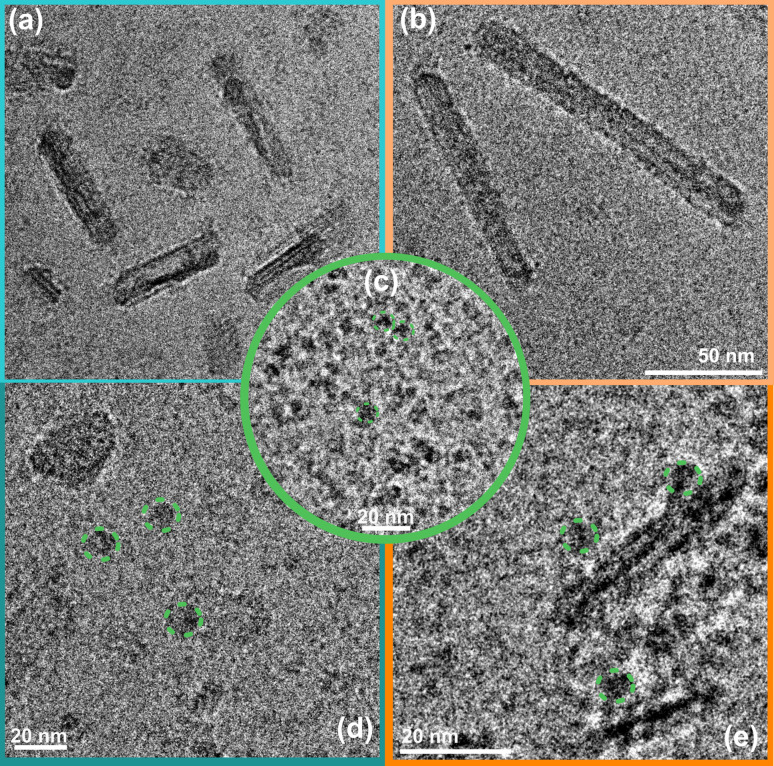
Cryo-EM micrographs of POZ-M(−) NRs (a), POZ-M(+) NRs (b) and the CODH enzyme (c) recorded under a CO_2_ atmosphere; and corresponding biohybrid assemblies after encapsulation with CODH for POZ-M(−) NRs (d), and POZ-M(+) NRs (e) respectively.

Cryo-EM image analysis suggests that the negatively charged organic NRs incubated with a CODH enzyme system (Fig. 3d and S9a†) show a lower tendency of the enzyme to be located near the POZ-M(−) NRs, furthermore supporting the electrophoresis data, where weak interaction for negatively charged NRs was observed. Nevertheless, as seen from Fig. 3e, and S9b† enzyme units are resting on the surface of positively charged POZ-M(+) NRs, in some cases covering the whole surface of NRs. From the relative dimensions of POZ-M(+) NRs and the CODH enzyme (Fig. S10,† 50 Å × 88 Å × 52 Å, determined from the crystal structure with PDB ID: 1SU6), we estimated that around 10–20 CODH units might bind to the surface of each organic NR. The close association between NRs and the enzyme is expected to have a positive impact on overall system performance.

**Fig. 4:**
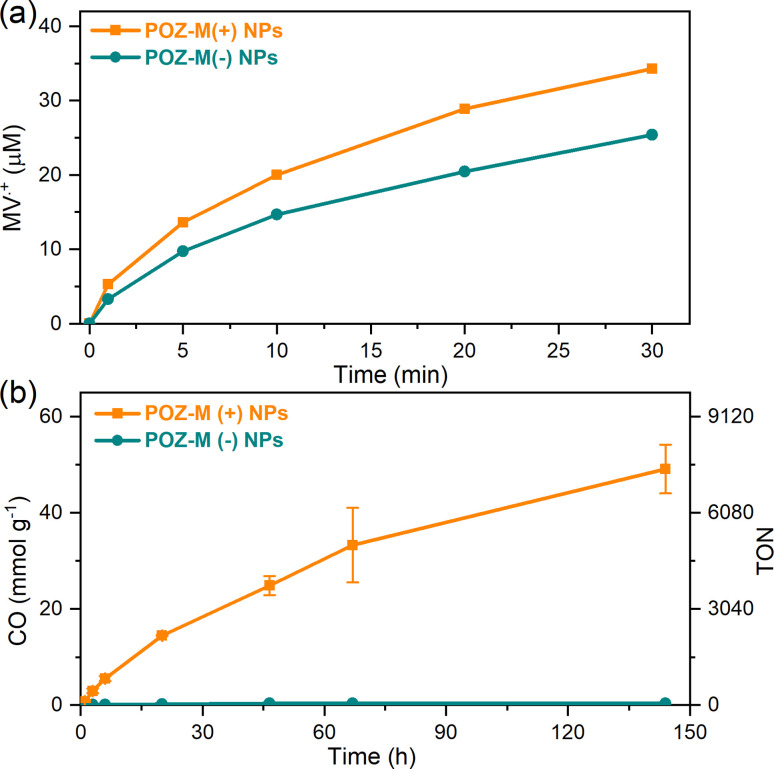
(a) Kinetics of methyl viologen photoreduction extracted at 605 nm for POZ-M(+) NRs and POZ-M(−) NRs illuminated over time under a CO_2_ atmosphere. (b) Photocatalytic data for POZ-M(+) NRs and POZ-M(−) NRs (38 μg mL^−1^) at pH 5.7 in the presence of 0.5 M cysteine, MV^2+^ (5 mM), and CODH enzyme (250 pmol). Reaction volume 2 mL.

The impact of surface charge on the capability of organic NRs to deliver electrons to CODH, as well as its role in overall CO_2_ conversion to CO was studied next in detail. In a photocatalytic reaction, we employed methyl viologen (MV^2+^) as an electron mediator to deliver electrons to CODH. To investigate the electron transfer suitability of synthesized organic NRs, a solution containing either POZ-M(−) NRs or POZ(+) NRs, with methyl viologen (MV^2+^) and cysteine was purged with CO_2_. After light illumination (LED, 420–750 nm, 50 mW cm^−2^), the rise of the characteristic reduced MV^2+^ (MV˙^+^) peak (605 nm, Fig. S11†) was monitored as a function of time (Fig. 4a). The corresponding reaction rates of MV^2+^ reduction (*ν*_NRs-MV_) by organic NPs in the presence of cysteine were estimated and are collected in Table S1.† By replacing the negatively charged POZ-M(−) NRs with positively charged POZ-M(+) NRs, we increased the rate of MV^2+^ reduction from *ν*_NRs-MV_ = 195 to 320 μmol L^−1^ h^−1^ (Fig. 4).

For CO_2_ photoreduction catalytic studies, biohybrid assemblies of POZ-M NRs and CODH enzyme were studied as follows. At first, vials containing organic nanoparticles and methyl viologen were gently mixed by CO_2_ purging inside the solution for 10 min. Afterwards, cysteine was introduced, and vials were sealed with rubber septa. The resulting mixture was purged with CO_2_ above the solution to prevent aggregation of nanoparticles for an additional 20 min (final pH 5.7). Finally, CODH was injected to the sealed vials inside the glovebox. The progress of CO production in the presence of biohybrid assemblies is presented in Fig. 4b (corresponding non-normalized data are presented in ESI, Fig. S12†). The photocatalytic activity of positively charged POZ-M(+) NRs was 54 mmol_CO_ g_NRs_^−1^ (TON = 8224, CO_2_ reduction rate 1140 μmol_CO_ g_NRs_^−1^ h^−1^), around two orders of magnitude higher than that for negatively charged POZ-M(−) NRs (0.33 μmol_CO_ g_NPs_^−1^, TON = 50).^30^ The resulting external quantum efficiency (EQE) of 0.22% was obtained for POZ-M(+) NRs at 450 nm (see the ESI†). The close interaction between POZ-M(+) NRs and the enzyme should be one of the factors responsible for the efficient photocatalysis of this system (more discussion below). In order to unveil the role of the surfactant we have additionally prepared organic NPs composed of the ABA surfactant (Fig. S13†). The photocatalytic activity of ABA NPs was five times lower than the activity of POZ-M(+) NRs, suggesting that the observed difference in activity between negatively charged POZ-M(−) NRs and positively charged POZ-M(+) NRs is not solely because of ABA addition, and is rather driven by a suitability of charged surface groups and morphology provided by POZ-M(+) NRs with respect to the CODH enzyme. In the absence and presence of the enzyme, POZ-M(+) NRs and POZ-M(−) NRs generated ∼1.2 μmol of hydrogen (Fig. S14†). It is worth pointing out that addition of CODH did not result in increased H_2_ formation, and additionally no other products were detected either in solution, or in the headspace, supporting the 100% selectivity of the CODH enzyme towards CO_2_ conversion into CO as proved by many other studies.^31^ In spite of 100% selectivity of the catalyst, the overall selectivity of the biohybrid system based on POZ-M(+) NRs is close to 63%. However, as the EQE of the system is relatively low, it is unlikely that the side reaction of H_2_ formation is impeding the flow of electrons to the CO_2_ reduction side. Once CO_2_ was replaced with Ar, no CO was formed for the POZ-M(+) NRs:CODH biohybrid assembly, suggesting that the origin of CO formation is indeed CO_2_. It is important to note that exclusion controls performed by removing individual components from the photocatalytic system resulted in negligible CO product formation (Fig. S15†), proving the photocatalytic reaction. A table comparing the performance of the biohybrid assembly POZ-M(+) NRs:CODH with that of the related state-of-the-art systems is presented in the ESI (Table S2†),^19–21,23,32–37^ from which one can see the first example of biohybrid assemblies based on organic photosensitizers and CODH, that is among the efficient systems considering both performance and stability, as well as the intrinsic activity of the utilized CODH enzyme.

**Fig. 5:**
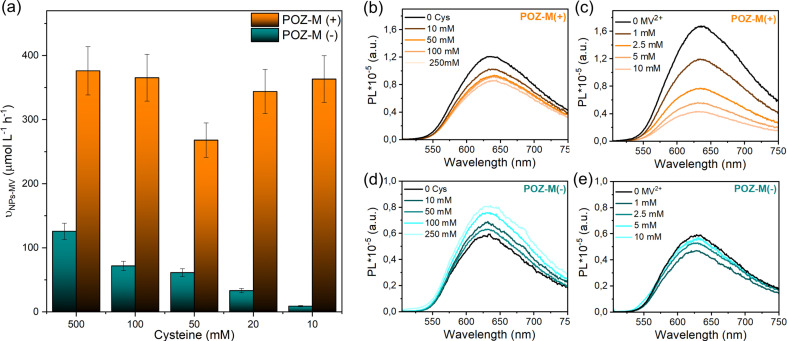
(a) Rates for reduced methyl viologen formation (*ν*_NPs-MV_) for POZ-M(+) NRs and POZ-M(−) NRs determined under Ar in the presence of various amounts of cysteine. Here, *ν*_NPs-MV_ values are determined during the first five minutes of light irradiation. Fluorescence quenching spectra of POZ-M(+) NRs (b, c) and POZ-M(−) NRs (d, e) with l-cysteine (10–250 mM) or methyl viologen (1–25 mM) under *λ*_exc._ = 495 nm.

In order to distinguish between the deactivation of the excited state of NPs *via* electron transfer from an electron donor (l-cysteine, reductive quenching) or *via* electron transfer to an electron acceptor (MV^2+^, oxidative quenching) we performed steady-state fluorescence quenching experiments (Fig. 5b and c). It was observed that 10 mM MV^2+^ gives 75% oxidative quenching of POZ-M(+) NR fluorescence, which is much more efficient than 16% reductive quenching given by 10 mM cysteine. This suggests that the excited POZ-M(+) NPs first reduce methyl viologen, and then the formed oxidized POZ-M NPs are regenerated by electron transfer from cysteine. Upon varying the concentration of cysteine (Fig. 5a), we observe little impact on the rate of MV^2+^ reduction for POZ-M(+) NRs. Notably, upon addition of cysteine into POZ-M(−) NR solution no quenching is observed; however the emission of POZ-M(−) NRs increases along with the increase in cysteine concentration. This phenomenon shows a strong indication that the cysteine significantly changes the local chemical environment of POZ-M(−) NRs. Since we did not see significant change in absorption spectra of POZ-M(−) NRs in the presence of cysteine (Fig. S16†), the POZ-M(−) NR core packing should not be changed much. This phenomenon can also be observed in the presence of MV^2+^ at a concentration of more than 1 mM. We therefore hypothesize that cysteine and MV^2+^ must have a strong interaction with POZ-M(−) NRs, but it is challenging to conclude the quenching process/mechanism of POZ-M(−) NRs in the presence of cysteine and MV^2+^. Nevertheless, the information obtained here is helpful for us to interpret the difference in photocatalytic performance between POZ-M(+) and POZ-M(−) NRs in the presence of the CODH enzyme, where limited electron transfer to the enzyme is observed for POZ-M(−) NRs. Concurrently, generation of reduced MV^2+^ by the negatively charged POZ-M(−) NRs is highly dependent on the concentration of cysteine (Fig. 5a).^40^

As mentioned in gel electrophoresis data and Cryo-EM (Fig. 3), positively and negatively charged POZ-M NRs have different interactions with the CODH enzyme. Armstrong *et al.* earlier highlighted that the photocatalytic activity of biohybrid assemblies was highly susceptible to the surface characteristics and shape of light harvesters that affected the co-attachment of the enzyme and the efficiency of electron transfer.^19^ To begin with POZ-M(+) NRs showed more efficient deactivation of the excited state *via* electron transfer to the redox mediator (Fig. 5b and c), in contrast to as prepared POZ-M NPs with a hollow morphology (Fig. S17a and b†). Furthermore, more efficient regeneration of POZ-M(+) NRs than in POZ-M(−) NRs at the same concentration of sacrificial electron donor, allowed the regeneration plateau to be achieved even with 10 mM cysteine (Fig. 5a). At the same time, even though negatively charged NRs are indeed able to give electrons to positively charged MV^2+^ (Fig. 5e), the generated MV˙^+^ most likely gets trapped within surfactant chains of POZ-M(−) NRs and does not reach the enzyme units that are located further away (Fig. S10†). Thus, the relatively superior photocatalytic activity of positively charged NRs must be driven by more favourable surface morphology that reduces MV^2+^ efficiently and has intimate interaction with CODH enzyme units, which allows the reduced MV^2+^ to facilely reach the CODH enzyme, therefore resulting in efficient charge transfer.

**Fig. 6:**
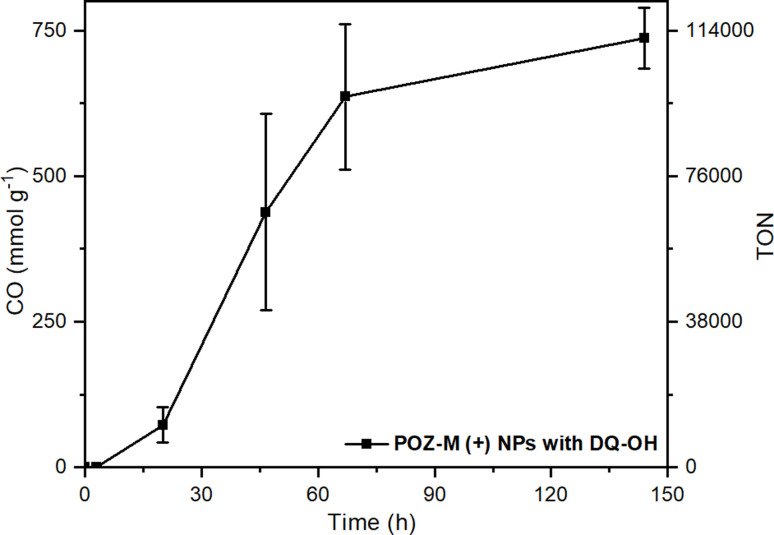
Photocatalytic data of POZ-M(+) NRs (38 μg mL^−1^) with DQ-OH (5 mM) as the redox mediator at pH 5.7 in the presence of 0.5 M cysteine and CODH enzyme (250 pmol). Reaction volume 2 mL.

In order to create future biohybrid assemblies one needs to understand the limiting factors of the already existing assemblies. The steady-state fluorescence quenching results indicate a rate determining step related to an electron transfer involving MV^2+^ rather than cysteine. To estimate if MV^2+^ reduction by the organic NRs or the electron transfer between MV˙^+^ and CODH is rate determining, we calculated the rates of both reactions (*ν*_NRs-MV_ and *ν*_MV-CODH_ respectively). In all cases both for POZ-M(+) NRs and POZ-M(−) NRs, *ν*_NRs-MV_ is much higher (320 and 195 μmol L^−1^ h^−1^) than *ν*_MV-CODH_ (41 and 0.8 μmol L^−1^ h^−1^), suggesting that the rate determining step of the photocatalysis is mainly the electron transfer from the redox mediator to the CODH enzyme. To confirm this, we have performed an additional photocatalytic experiment by replacing MV^2+^ with the diquat derivative abbreviated as DQ-OH (7-hydroxy-3,11-dimethyl-7,8-dihydro-6*H*-dipyrido[1,2-*a*:2′,1′-*c*][1,4]diazepine-5,9-diium), which provides 140 mV more reducing driving force than MV^2+^.^38^ By replacing MV^2+^ with DQ-OH with a more negative reduction potential we have increased the TOF to 0.6 s^−1^ and TON from 8224 to 120 000 (Fig. 6). This supports the earlier suggestion that the rate limiting step of the POZ-M(+) NRs:CODH is the electron transfer from the reduced MV˙^+^ to CODH. However, one should remember that the driving force between the redox mediator and CODH is not the only parameter that determines the system's performance, as reported recently by Kim *et al.*, and further work is necessary.^39^ Furthermore, after using the best performing biohybrid assembly based on POZ-M(+) NRs and replacing MV^2+^ with DQ-OH we could achieve better efficiency and improve the product selectivity of CO up to 96%.

## Conclusions

In summary, organic nanorods (NRs) self-assembled by using a small organic phenoxazine dye (POZ-M) have been used to drive photobiocatalytic CO_2_ reduction into CO using CODH as the biocatalyst. The positively charged POZ-M(+) NRs prepared with the ABA amphiphilic polymer surfactant showed superior photocatalytic activity to negatively charged POZ-M(−) NRs covered with PEG by 2 orders of magnitude. The performance and selectivity of the biohybrid assembly could be further enhanced by replacing MV^2+^ with DQ-OH that provides more driving force for enzyme reduction (TON 120 000). From the electrophoresis experiment, photophysical study and Cryo-EM morphology analysis studies, it is suggested that positively charged POZ-M(+) NRs with favourable surface charge and elongated morphology have intimate interaction with all components used in photocatalysis, which is therefore beneficial for having an enhanced photocatalytic performance as compared to the negatively charged POZ-M(−) NRs. This work demonstrates a new straightforward and universal strategy of small organic molecular nanoparticle functionalisation with suitable surfactants in water to match the needs of the biocatalyst and points out the importance of surface charges of the nanoparticle in the interaction between the artificial photosensitizer and the bio-catalyst, that can be successfully translated for developing future bio-hybrid photocatalytic systems for solar fuel and solar chemical production.

## Data availability

The data supporting this article have been included as part of the ESI.† The raw data are available upon request from the authors.

## Author contributions

The manuscript was written through contributions of all authors.

## Conflicts of interest

There are no conflicts to declare.

## Supplementary Material

SC-OLF-D4SC03154G-s001
