# Supplementary material for: Photobiocatalytic CO2 reduction into CO by organic nanorod-carbon monoxide dehydrogenase assemblies: surfactant matters
Source: Chem Sci. 2024 Sep 18;15(40):16789–95. doi: 10.1039/d4sc03154g (PMC11421036; doi:10.1039/d4sc03154g)
Supplement: SC-015-D4SC03154G-s001 [file SC-015-D4SC03154G-s001.pdf]

Supplementary Information (SI) for Chemical Science.
This journal is © The Royal Society of Chemistry 2024

# Supporting Information:

# Photobiocatalytic $CO_2$ Reduction into CO by Organic Nanorods-Carbon Monoxide Dehydrogenase Assemblies: Surfactant Matters

Mariia V. Pavliuk,[a] Maximilian Böhm,[b] Janna Wilhelmsen,[a] Steffen Hardt,[c] Henrik Land[b] and Haining Tian*[a]

[a] Department of Chemistry - Ångström Laboratory, Physical Chemistry, Uppsala University, 751 20 Uppsala, Sweden.
[b] Department of Chemistry - Ångström Laboratory, Molecular Biomimetics, Uppsala University, 751 20 Uppsala, Sweden.
[c] Leiden Institute of Chemistry, Energy and Sustainability - Catalysis and Surface Chemistry, Leiden University, Einsteinweg 55, 2333 CC Leiden, The Netherlands

E-mail: haining.tian@kemi.uu.se


| Table of Content | Page |
|---|---|
| **I. Experimental section** | S2 |
| **Figure S1.** Size distribution of POZ-M(+) NPs and POZ-M(-) NPs determined with dynamic light scattering. | S7 |
| **Figure S2.** Zeta potentials (ξ) for as-synthesized POZ-M(+), POZ-M(-) NPs and under photocatalytic conditions ($CO_2$ atmosphere, pH 5.7). | S8 |
| **Figure S3.** Cryo-EM micrographs of as-synthesized POZ-M(-) NPs, POZ-M(+) NPs. | S9 |
| **Figure S4.** Cryo-EM micrographs of POZ-M(-) NRs, POZ-M(+) NRs under Ar atmosphere. | S10 |
| **Figure S5.** Normalized UV-Vis absorption and steady-state photoluminescence spectra for small molecule POZ-M in THF. | S11 |
| **Figure S6.** Non-normalized steady-state UV-Vis absorption of POZ-M (+) NRs and POZ-M (-) NRs | S12 |
| **Figure S7.** Steady-state PL spectra of organic NPs (POZ-M (+) NRs with ABA surfactant, POZ-M NRs without ABA surfactant and NPs based on ABA surfactant) upon excitation at 420 nm. | S13 |

| | |
|---|---|
| **Figure S8.** Zeta potentials (ξ) for POZ-M(+), POZ-M(-) NRs before and after incubation with the CODH enzyme under photocatalytic conditions ($CO_2$ atmosphere, pH 5.7) | S14 |
| **Figure S9.** Cryo-EM micrographs of biohybrid assemblies between POZ-M(-) NRs, POZ-M(+) NRs with CODH enzyme recorded under $CO_2$ atmosphere. | S15 |
| **Figure S10.** Cryo-EM micrographs of CODH enzyme and Cryo-EM grids without any substrate. | S16 |
| **Table S1.** Rates for reduced methyl viologen formation ($\boldsymbol{\upsilon}_{\mathbf{NRs\text{-}MV}}$) and rates of reduced CODH formation ($\boldsymbol{\upsilon}_{\mathbf{MV\text{-}H2ase}}$) in the presence of various organic NPs | S17 |
| **Figure S11.** Generation of reduced methyl viologen for (a) POZ-M(+) and (b) POZ-M(-) NRs during light irradiation. | S17 |
| **Figure S12.** Photocatalytic data for (a) POZ-M(+) NRs and POZ-M (-) NRs (38 µg/mL) with $MV^{2+}$ (5 mM), and for (b) POZ-M(+) NRs with DQ-OH (5mM) at pH 5.7 in the presence of 0.5 M cysteine and CODH enzyme (250 pmol). | S18 |
| **Figure S13.** (a) Steady-state UV-Vis absorption of ABA NPs. (b) Photocatalytic data for ABA NPs and POZ-M(+) NRs at pH 5.7 in the presence of cysteine, $MV^{2+}$, and CODH enzyme under $CO_2$ atmosphere. | S19 |
| **Figure S14.** Photocatalytic data for POZ-M(+) NRs and POZ-M(-) NRs (38 µg/mL) at pH 5.7 in the presence of 0.5 M cysteine, MV2+ (5 mM), and CODH enzyme (250 pmol). | S20 |
| **Figure S15.** Blank photocatalytic data for various Pdots and small molecule NPs | S21 |
| **Figure S16.** UV-Vis spectra for POZ-M(+) NRs(a,b) and POZ-M(-) NRs (c,d) with L-cysteine or methyl viologen. | S22 |
| **Figure S17.** Fluorescence quenching spectra for POZ-M(+) NPs. | S23 |
| **Table S2.** The Table comparing the performance of POZ-M(+) NRs with the related state-of the art systems. | S24 |
| **References** | S25 |


# I. Experimental section.

**Materials.** POZ-M was synthesized using a synthetic procedure described in ref. [1]. F8T2 polymer (Mw 64 kDa, [poly(9,9-dioctylfluorene-alt-bithiophene)]) was purchased from Ossila, U.K. PFODTBT polymer (Mw 50-80 kDa, [poly((9,9-dioctylfluorenyl-2,7-diyl)-alt-5,5-(40,70-di-2-thienyl-20,10,30-benzothiadiazole))] was purchased from Solaris Chem. PEG copolymer (polystyrene grafted with carboxy terminated polyethylene oxide, backbone chain Mw 8500, graft chain Mw 4600, total chain Mw 36.5 kDa)) and triblock copolymer ABA (Mw 20−30 kDa, [poly(N,N-dimethylamino ethyl

methacrylate)-B-poly(9,9-N-dihyxyl-2,7-fluorene)-B-poly(N,N-dimethylamino ethyl methacrylate)]) were purchased from Polymer Source Inc., Canada. The centrifugal filters with Mw cutoff of 10 kDa (Amicon Ultra-15) were purchased from Sigma-Aldrich. The syringes filters with 0.45 µm pore size and PVDF membrane have been purchased from PALL (Pall corporation). Tetrahydrofuran (THF) was purchased from VWR Chemicals. All the chemicals were used without any further filtration or purification unless stated otherwise.

**Preparation of Small Molecule Organic Nanoparticles (POZ-M NRs).** NPs were synthesized using a modified nanoprecipitation method. At the end of each procedure NPs were concentrated using centrifuge filters. The final concentration of organic molecules in NPs was determined by analyzing the absorbance spectra of the freeze-dried NPs redissolved in THF. POZ-M was dissolved in THF at a concentration of 1 mg/mL and then sonicated for 5 min. Negatively charged surfactant PEG (4.5 mg/mL) and positively charged surfactant ABA (2.25 mg/mL) were dissolved in THF and sonicated for 25 and 5 min respectively. Then solutions were mixed in different ratios with addition of THF. In order to obtain POZ-M(-) NPs ratio of POZ-M:PEG was 1: 9 ($Vt_{otal}$ = 500 µL). In order to obtain POZ-M(+) NPs ratio of POZ-M:ABA was 1: 9 ($Vt_{otal}$ = 500 µL). These mixtures were sonicated for ten minutes and then injected fast to a separate vial with 12 mL of deionized water. Resulting solutions were covered with an aluminum foil to avoid exposure of light, and were sonicated for additional 6-9 min. THF was removed by slow evaporation overnight. Resulting NPs were filtered with a 0.45 µm syringe filter. Furthermore, corresponding POZ-M(-) and POZ-M(+) nanorods were prepared by purging either $CO_2$ or Ar for 10 min.

**Preparation of *Ch*-CODH-II.** The *coos2* gene from *Carboxydothermus hydrogenoformans* (WP_011343033.1) was synthesized and cloned in pET-11a(+) with a C-terminal StrepII-tag by Genscript® using restriction sites NdeI and BamHI following codon optimization for expression in *E. coli*. The construct was expressed in *E. coli* DE3 BL21 Δ*iscR*. The cells were grown in LB-medium (with 0.1 M phosphate buffer pH 7.4, 1 mM $NiCl_2$, 1 mM iron citrate, 4 mM L-cysteine, 0.5 % glucose, 100 µg/ml ampicillin sodium salt, 50 µg/ml kanamycin monosulfate) under shaking at 37 °C to an $OD_{600}$ of 0.4. Antifoam C (200000x dilution) was added and the cells where bubbled with nitrogen gas for at least 30 min to remove oxygen. Under nitrogen bubbling, IPTG was added to a final concentration of 200 µM, to induce expression. The cells were incubated for 20 h at 30 °C under shaking. The cells where harvested, transferred to an anoxic environment and incubated with lysis buffer (50 mM Tris,

pH 8, 20 mM NaCl, 2 mg/ml lysozyme, 0.1 mg/mL DNAse, 0.1 mg/mL RNAse, 0.1 % sodium desoxycholate, 1 % saccharose, 1 mM sodium dithionite) for 30 min at room temperature followed by sonication. The soluble fraction was separated from the insoluble residue via ultra-centrifugation. The protein of interest was extracted from the supernatant via a Strep-Tactin XT column from Cytiva. The protein was eluted in 50 mM Tris, pH 8, with 5 % glycerol, 20 mM NaCl, 50 mM biotin, concentrated and stored at -80 °C. Protein concentration was determined using the Bradford assay. The iron and nickel content per monomer where determined to be 9 and 1, respectively, via ICP-OES. The specific activity of $CO_2$ reduction of the purified *Ch*-CODH-II was determined to be 0.12 μmol CO $min^{-1}$ $mg^{-1}$ enzyme at pH 6.5, under literature assay conditions.[2]

**General methods.** The hydrodynamic diameters and surface ξ-potentials were conducted on a Zetasizer Nano S (Malvern, U.K) and a Zetasizer Nano-ZS (Malvern, U.K.) in folded capillary zeta cells, respectively.

***The UV-Vis absorption spectra*** of the synthesized NRs were recorded using a Varian Cary 50. The steady-state fluorescence and excitation spectra have been recorded on Flurolog iHR 320 (Horiba Jobin Yvon) using the Fluoracle software (the absorbance of NRs was adjusted to 0.05 a.u.). Both steady-state absorption and fluorescence spectra were recorded in quartz cuvettes. The photoluminescence quantum yield at 495 nm was estimated using Coumarin 343 in ethanol solvent as reference (63%).

***Electrophoretic mobility shift assay.*** The protein (25 µM final concentration) was incubated with and without POZ-M(-) and POZ-M(+) at room temperature for 1 h in the dark. Afterwards loading buffer was added and 10 µL of sample was loaded on a 1 % agarose gel and run at 75 V for several hours. During that time the propagation of the nanoparticles was tracked via their UV emission. The 1 % agarose gels were prepared and ran in either pH 8.6 TAE buffer (for POZ-M(-)) or pH 7 TAE buffer (for POZ-M(+)) with same ionic strength (60 mM) and different ratios of tris base and acetic acid. The gel was afterwards rinsed with water, fixated with 20:10:70 EtOH:Acetic acid:water for 30 min, and stained with PageBlue Protein Staining Solution (Thermo Scientific) for 30 min, and destained with water over night.

***Cryo-Transmission Electron Microscopy (Cryo-EM)*** has been performed on a Zeiss Libra 120 transmission electron microscope (Carl Zeiss AG, Oberkochen, Germany) that is operating at 80 kV and in zero-loss bright-field mode. To obtain digital images low-dose conditions with a BioVision Pro-SM Slow Scan CCD camera (Proscan Elektronische Systeme GmbH, Scheuring, Germany) were used. R1.3/1.2 200 and 300 mesh grids (QuantiFoil) were glow-discharged (20 mA for 120 s) on a PELCO EasiGlow. Concentrations of POZ-M(-) and POZ-M(+) NRs were 300 µg/mL. Each mixture (3 µL) was applied onto grids before plunge-freezing into liquid ethane in a Vitroblot Mark IV robot (FEI/Thermo Fisher Scientific) operating at 25 °C, 95 % humidity (blot time of 4 s). Samples were vitrified in liquid ethane and transferred to the microscope, continuously kept below –160 °C and protected against atmospheric conditions.

***Photocatalytic studies.*** The photocatalytic performance of organic nanoparticles with CODH enzyme has been studied in sealed vials (total volume 9 mL) under $CO_2$ atmosphere. As a rule, organic nanoparticles were at first gently mixed with methyl viologen by $CO_2$ purging inside the solution for 10 min. Afterwards, 0.5 M cysteine (pH 6.7) has been introduced and resulting mixture was purged with $CO_2$ to reach saturation for additional 20 min above the

solution to prevent aggregation. CODH enzyme has been injected to the sealed vials inside the glovebox. Photocatalytic mixtures were illuminated with a LED lamp (17 W, 420-750 nm, 50 mW $cm^{-2}$), and the produced CO was measured using gas chromatography (Thermo Scientific TRACE 1300, Italy). Gaseous products were analyzed extracting 100 μL of headspace from the photocatalytic vials with the gas-tight syringe at specific time points and injecting it to a GC. In order to prevent oxygen leakage during the gas extraction with the gas-tight syringe, the cap of the photocatalytic vial was covered with glue like clay Play-Doh (Hasbro Inc). All experiments were repeated in triplicates.

We have estimated external quantum efficiency (EQE) according to eq. 1.

$$EQE\ (\%) = 2\frac{n\ (CO)\cdot N_A\cdot h\cdot c}{(t_{irr}\cdot \lambda\cdot I\cdot A)} \quad \text{(eq. 1)},$$

where *n(CO)* is the moles of photogenerated carbon monoxide, $N_A$ is the Avogadro constant, *h* is the Planck constant, *c* is the speed of light, λ is the excitation wavelength, $t_{irr}$ is the irradiation time, *I* is the intensity of illumination, A is the irradiated area. Samples were illuminated with Xe lamp (300 W, AULTT CEL-HXF300 / CEL-HXUV300) equipped with an AM1.5 filter and bandpass filter (CEAULIGHT, 450 nm).

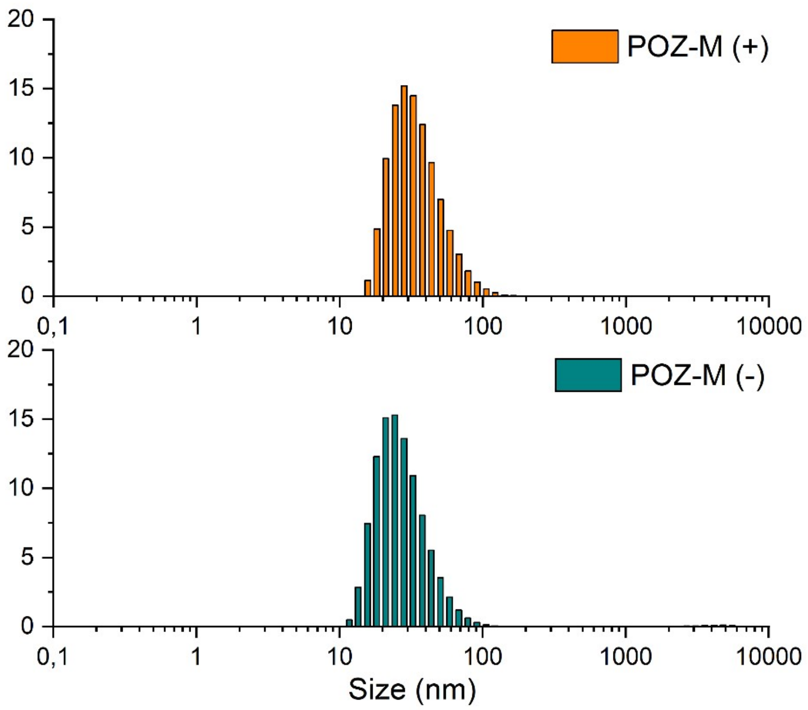


**Figure S1.** Size distribution of POZ-M(+) NPs and POZ-M(-) NPs determined with dynamic light scattering.

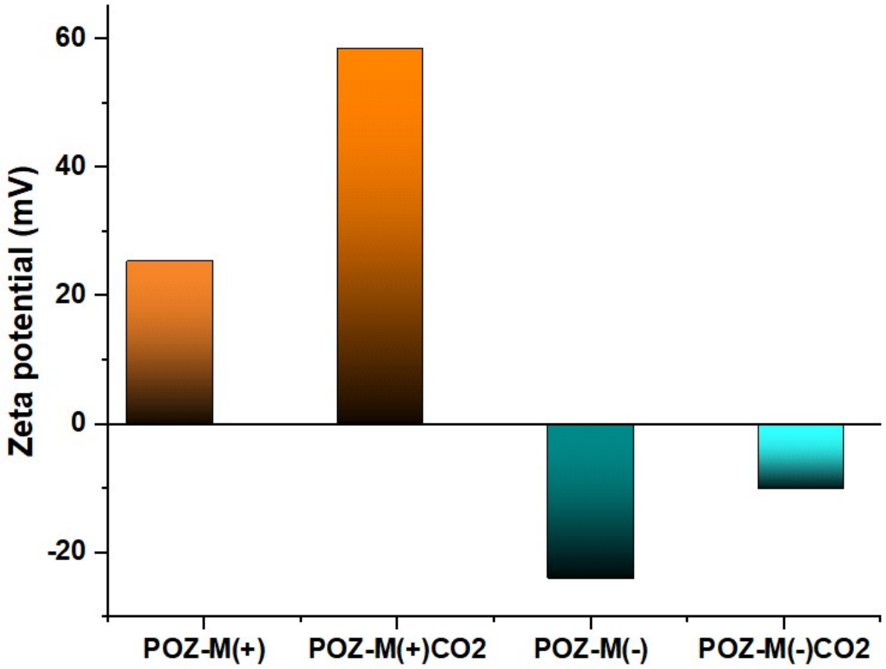


**Figure S2.** Zeta potentials (ξ) for as-synthesized POZ-M(+), POZ-M(-) NPs and under photocatalytic conditions ($CO_2$ atmosphere, pH 5.7)

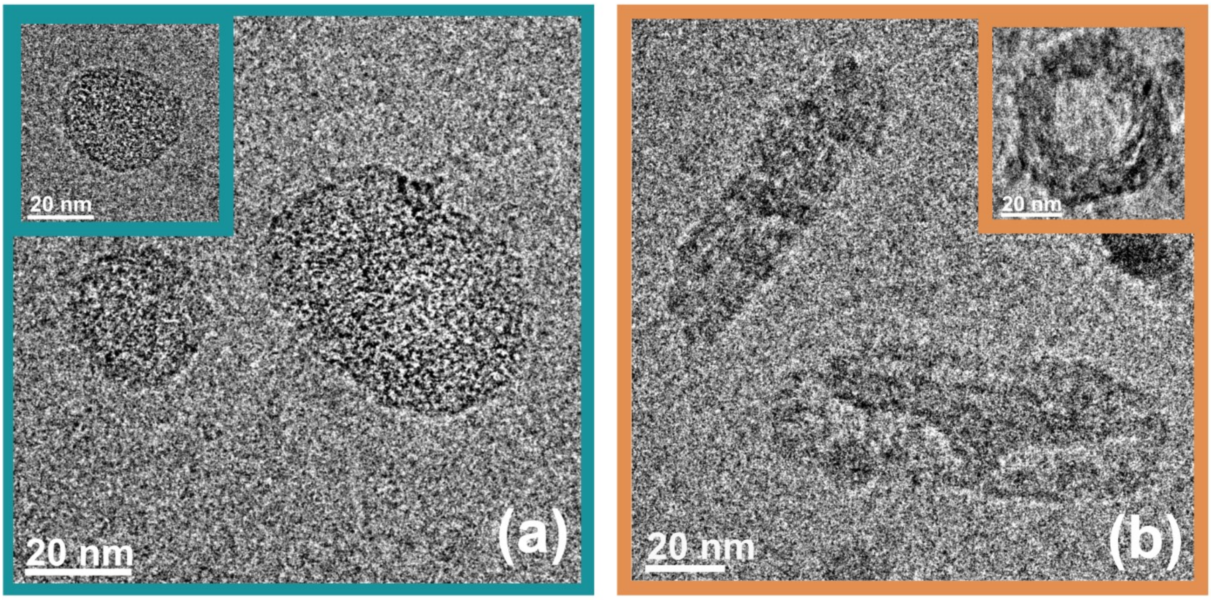


**Figure S3.** Cryo-EM micrographs of as-synthesized POZ-M(-) NPs (a), POZ-M(+) NPs.

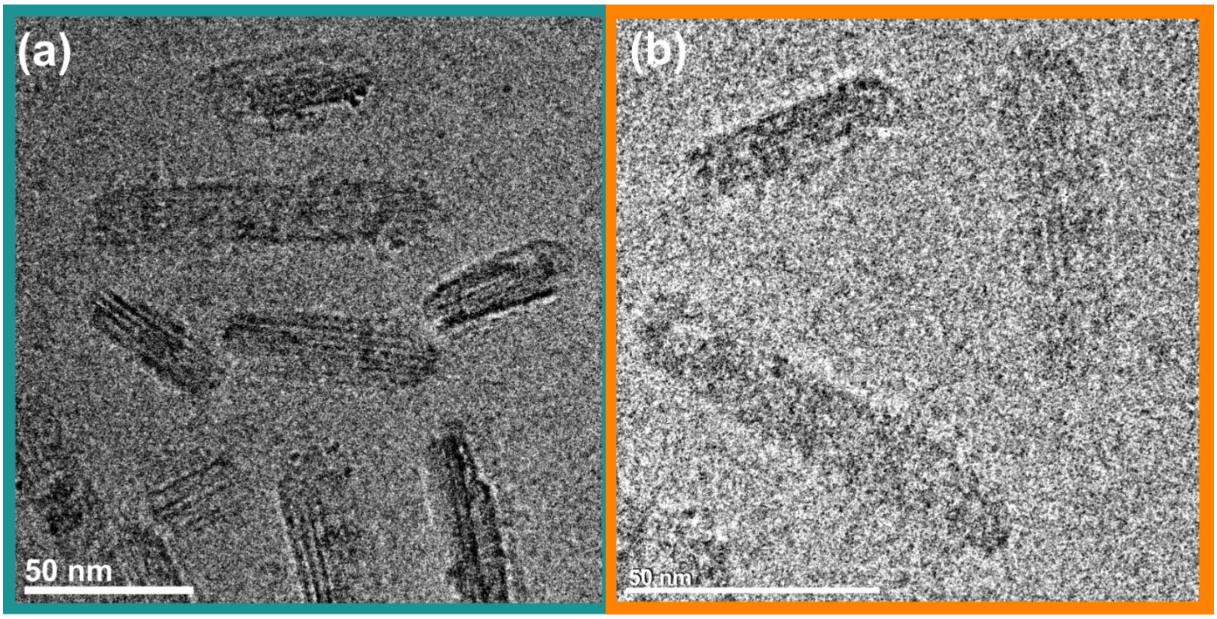


**Figure S4.** Cryo-EM micrographs of POZ-M(-) NRs (a), POZ-M(+) NRs (b) under Ar atmosphere.

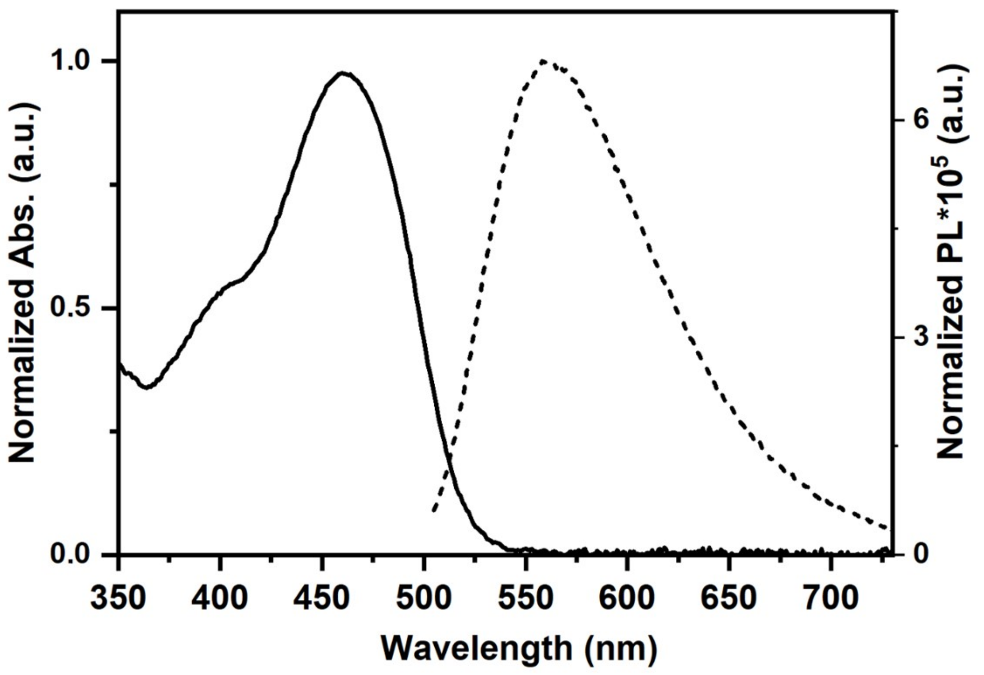


**Figure S5.** Normalized UV-Vis absorption (solid line) and steady-state photoluminescence (dashed line) spectra for small molecule POZ-M in THF.

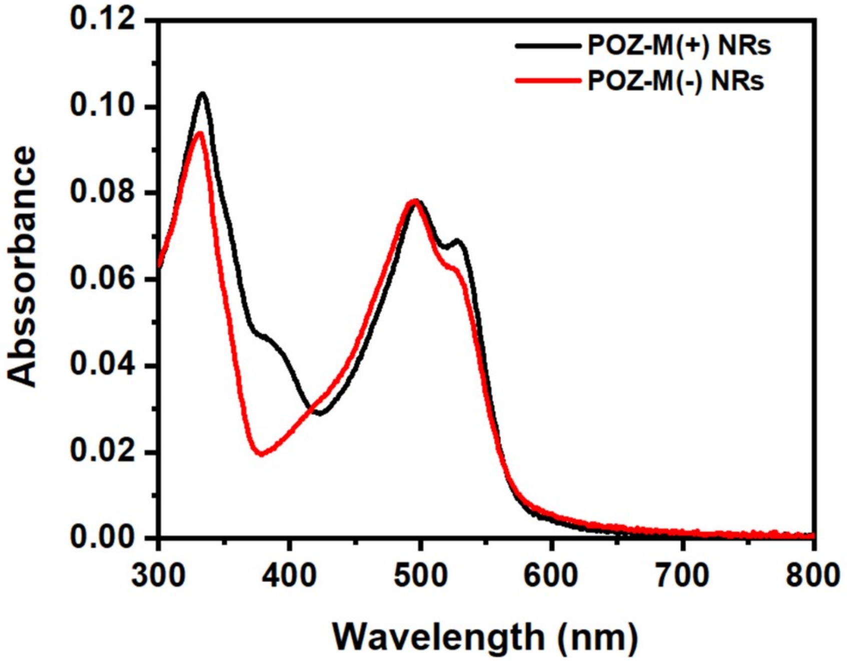


**Figure S6.** Non-normalized steady-state UV-Vis absorption of POZ-M (+) NRs and POZ - M( - ) NRs

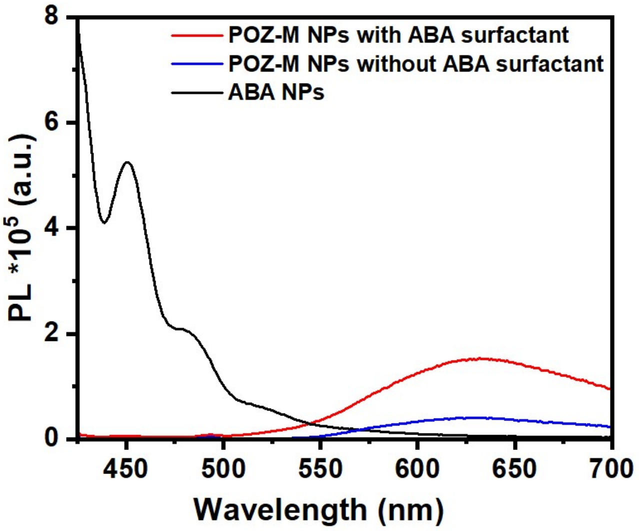


**Figure S7.** Steady-state PL spectra of organic NPs (POZ-M (+) NRs with ABA surfactant, POZ-M NRs without ABA surfactant and NPs based on ABA surfactant) upon excitation at 420 nm.

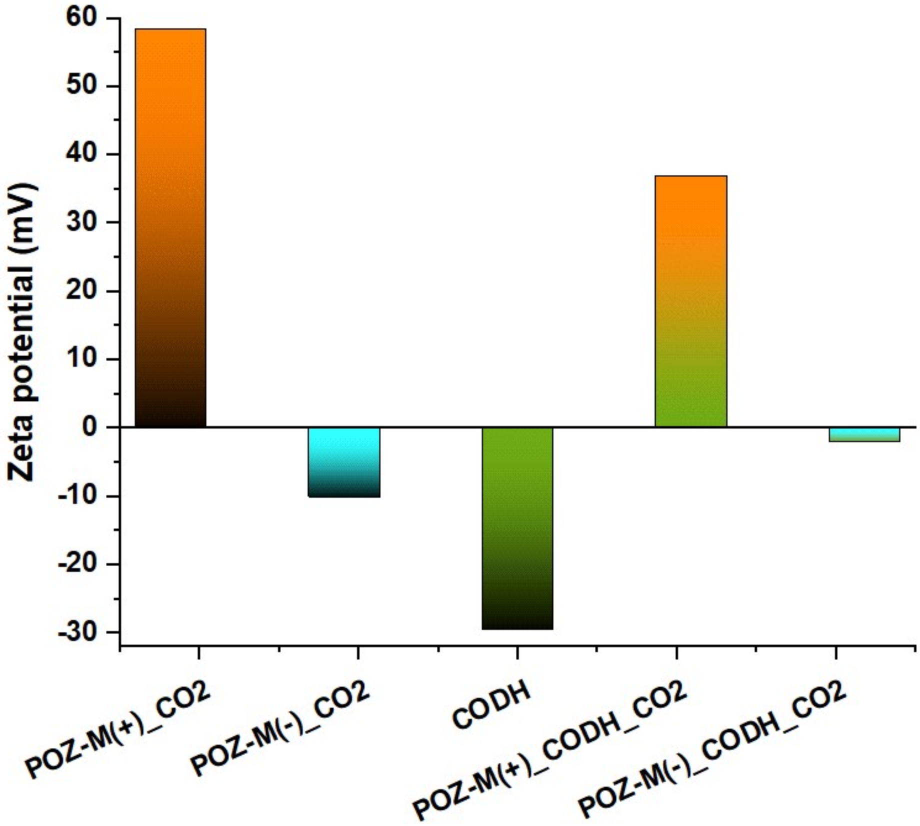


**Figure S8.** Zeta potentials (ξ) for POZ-M(+), POZ-M(-) NRs before and after incubation with the CODH enzyme under photocatalytic conditions ($CO_2$ atmosphere, pH 5.7)

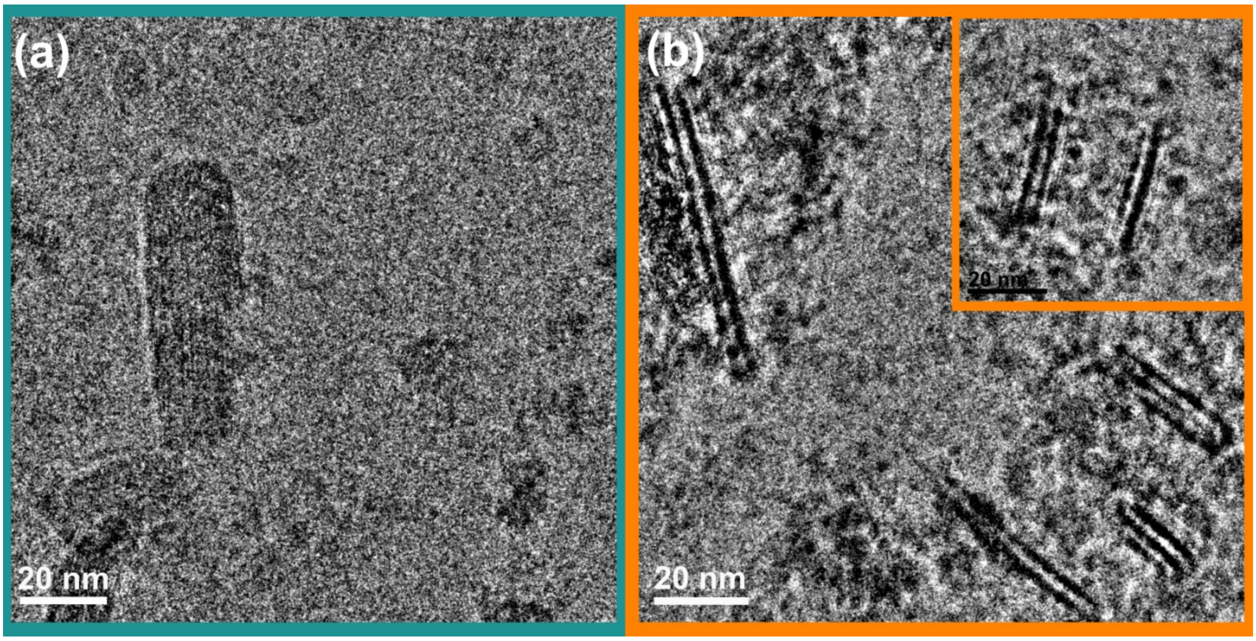


**Figure S9.** Cryo-EM micrographs of biohybrid assemblies between POZ-M(-) NRs (a), POZ-M(+) NRs (b) with CODH enzyme recorded under $CO_2$ atmosphere.

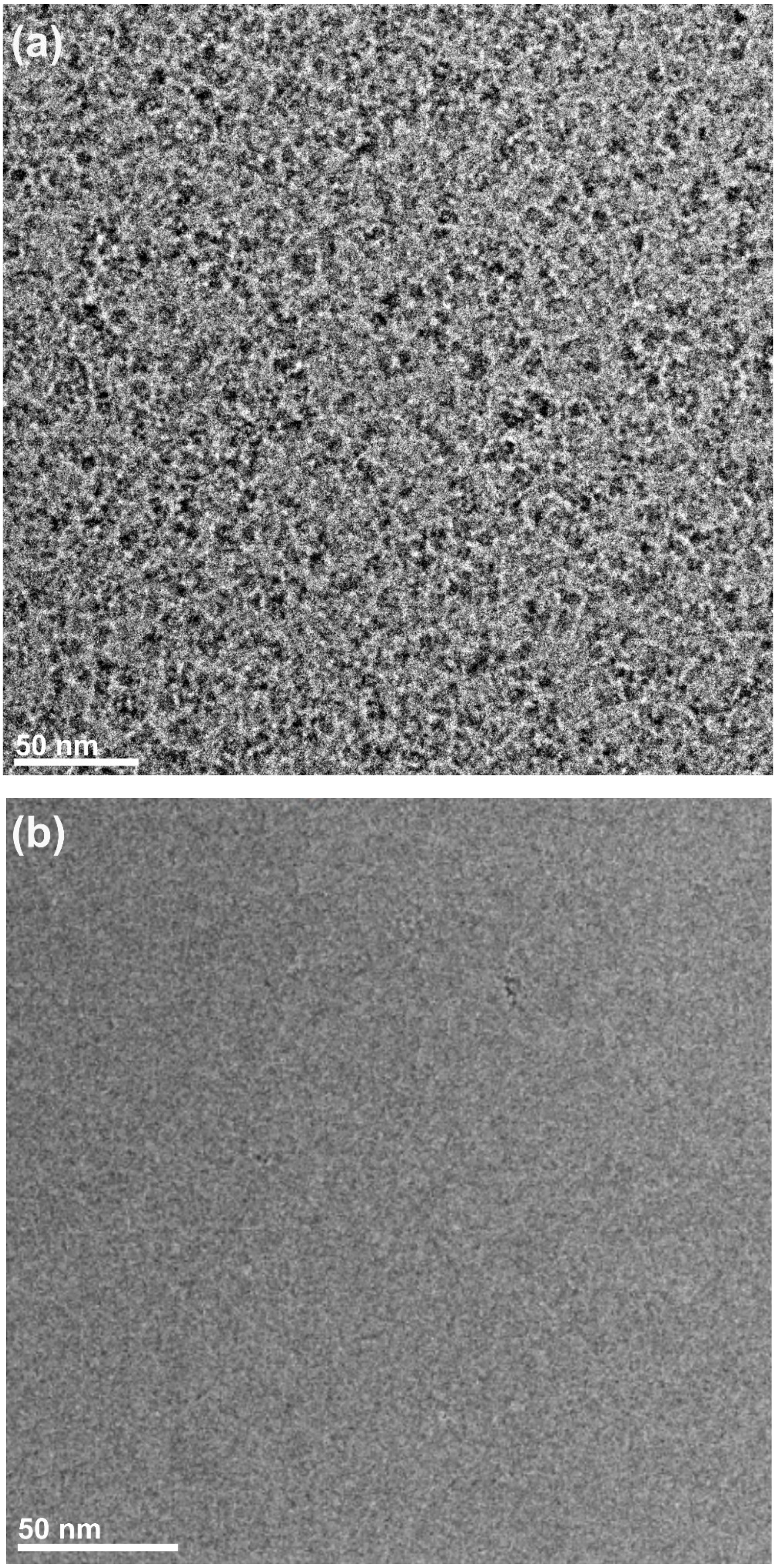

**Figure S10.** Cryo-EM micrographs of CODH enzyme and grids without any substrate.

**Table S1.** Rates for reduced methyl viologen formation (**$\upsilon_{NRs\text{-}MV}$**) and rates of reduced CODH formation (**$\upsilon_{MV\text{-}CODH}$**) in the presence of various organic NPs. **$\upsilon_{NRs\text{-}MV}$** values are determined as an amount of produced $MV^{\bullet +}$ ($\varepsilon$ = 13700 $M^{-1} \cdot cm^{-1}$ at 605 nm) during the first minute of light irradiation under $CO_2$ atmosphere with POZ-M 9 µg/mL (in parentheses **$\upsilon_{NRs\text{-}MV(max)}$** values are reported as the maximum observed rates during first 30 min of light irradiation). **$\upsilon_{MV\text{-}H2ase}$** values are determined from the amount of evolved CO during the first hour of light irradiation with POZ-M 19 µg/mL

| **Sample** | **$\upsilon_{NRs\text{-}MV}$ (µmol·$L^{-1}h^{-1}$)** (under $CO_2$, pH 5.7) | **$\upsilon_{MV\text{-}CODH}$ (µmol·$L^{-1}h^{-1}$)** (under $CO_2$, pH 5.7) |
|---|---|---|
| POZ-M (+) NRs | 320 (max 320) | 23 (max 41) |
| POZ-M (-) NRs | 195 | 0.8 |

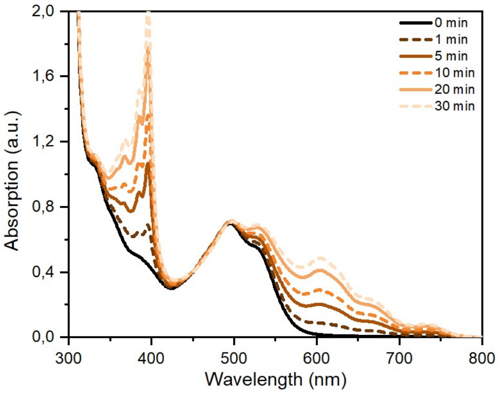


(a)

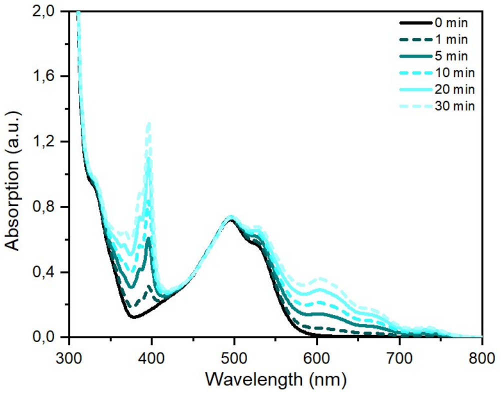


(b)

**Figure S11.** Generation of reduced methyl viologen for (a) POZ-M(+) and (b) POZ-M(-) NRs during light irradiation under photocatalytic conditions (pH 5.7, $CO_2$ atmosphere).

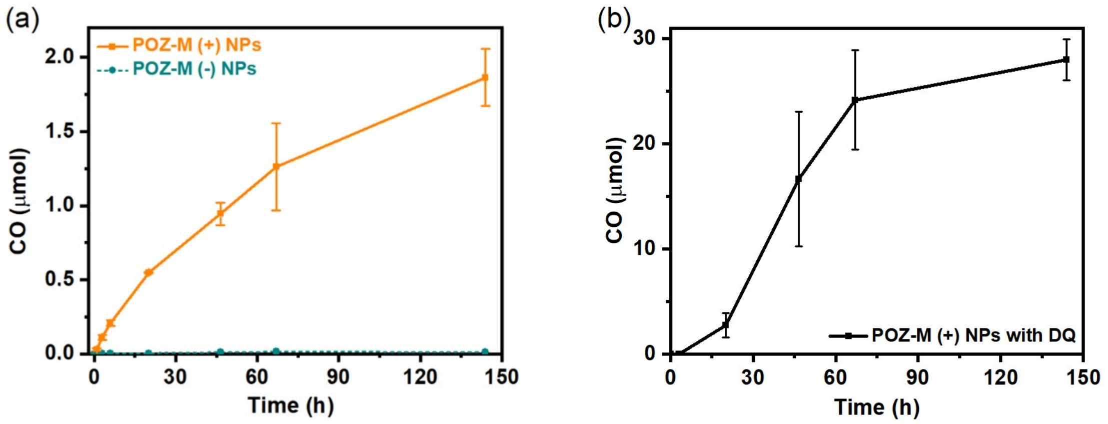


**Figure S12.** Photocatalytic data without normalization with catalyst weight and volume for (a) POZ-M(+) NRs and POZ-M (-) NRs (38 μg/mL) with $MV^{2+}$ (5 mM), and for (b) POZ-M(+) NRs with DQ-OH (5mM) at pH 5.7 in the presence of 0.5 M cysteine and CODH enzyme (250 pmol), initiated by LED irradiation (50 mW $cm^{-2}$, 420–750 nm). Total reaction volume 2 mL.

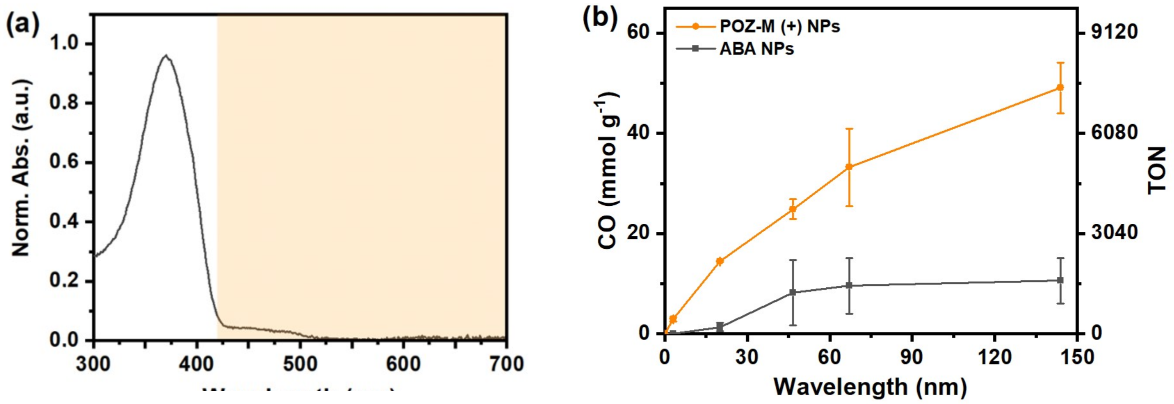


**Figure S13.** (a) Steady-state UV-Vis absorption of ABA NPs. In yellow we highlighted the area covered by LED lamp. (b) Photocatalytic data for ABA NPs (grey line) and POZ-M(+) NRs (orange line) at pH 5.7 in the presence of 0.5 M cysteine, $MV^{2+}$ (5 mM), and CODH enzyme (250 pmol) under $CO_2$ atmosphere.

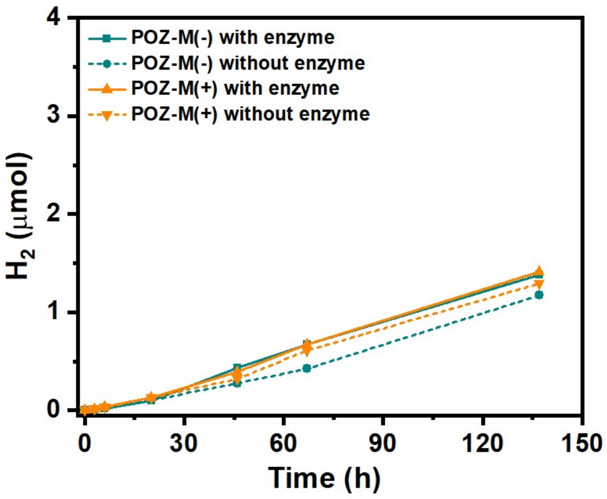


**Figure S14.** Photocatalytic data for POZ-M(+) NRs and POZ-M(-) NRs (38 µg/mL) at pH 5.7 with (solid lines) and without (dashed lines) CODH enzyme (250 pmol) in the presence of 0.5 M cysteine, $MV^{2+}$ (5 mM), initiated by LED irradiation (50 mW $cm^{-2}$, 420–750 nm). Total reaction volume 2 mL.

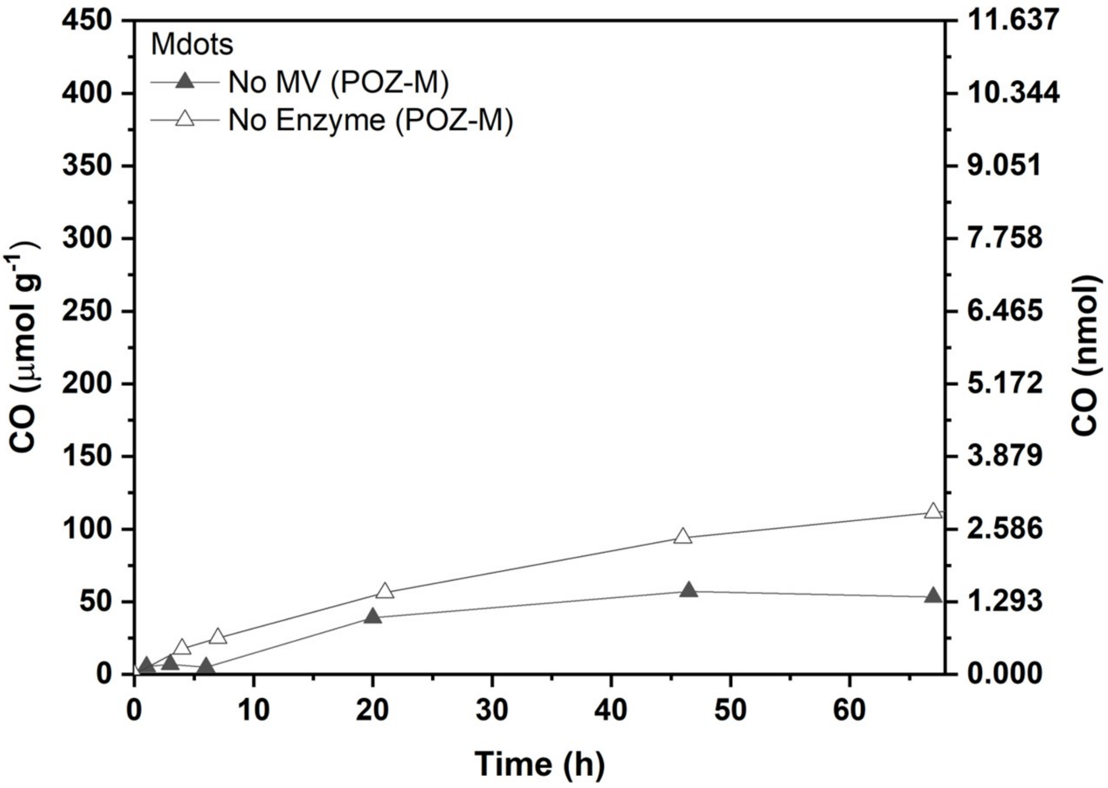


**Figure S15.** Blank photocatalytic data for small molecule POZ-M (+) NPs in the absence of methyl viologen or CODH enzyme initiated by LED irradiation (420-750 nm, 50 mW·cm$^{-2}$)[3] obtained inside headspace vials (Chromacol™, 6 mL headspace) under ambient conditions 30 °C. The results obtained without light harvesting organic NRs revealed negligible product formation.

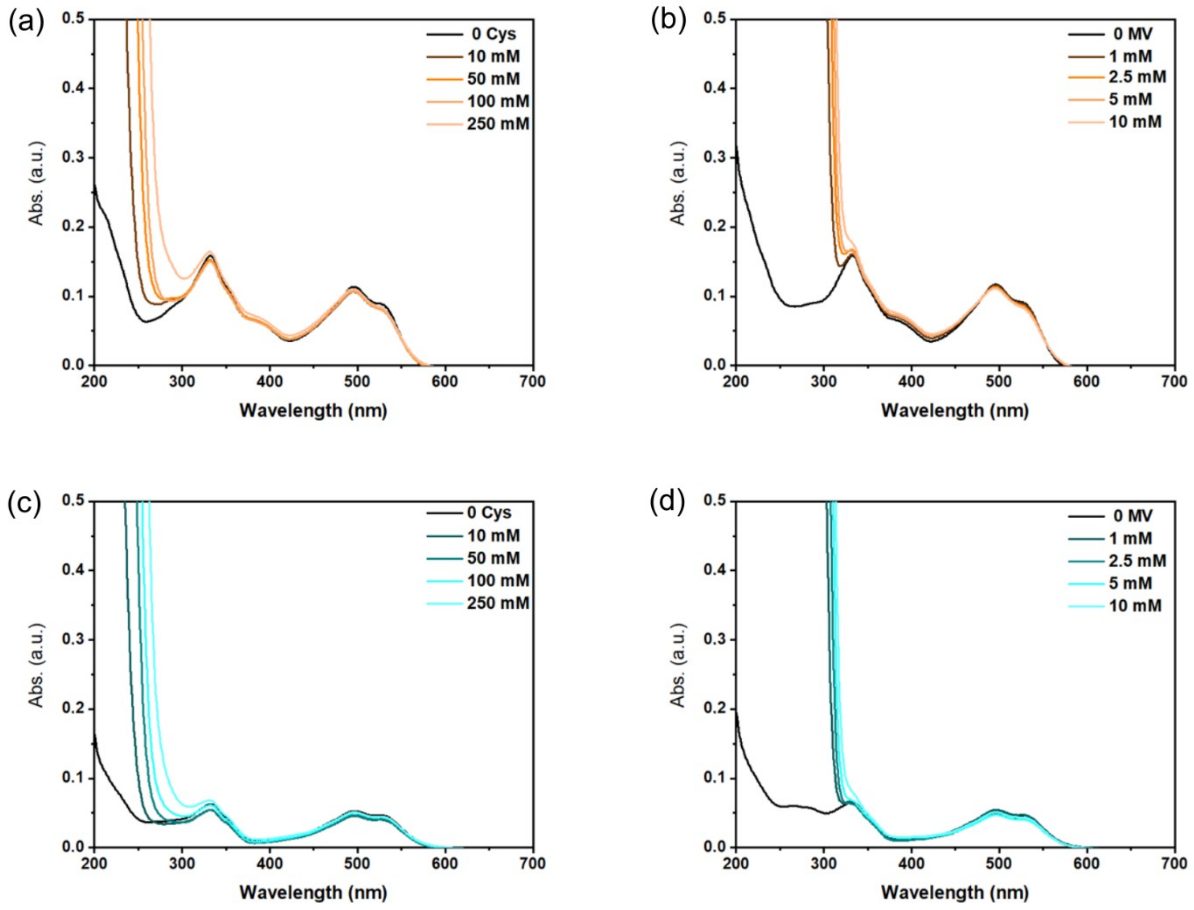


**Figure S16.** UV-Vis spectra for POZ-M(+) NRs(a,b) and POZ-M(-) NRs (c,d) with L-cysteine (10−250 mM) or methyl viologen (1−25 mM) used for the fluorescence quenching studies of nanorods under photocatalytic conditions.

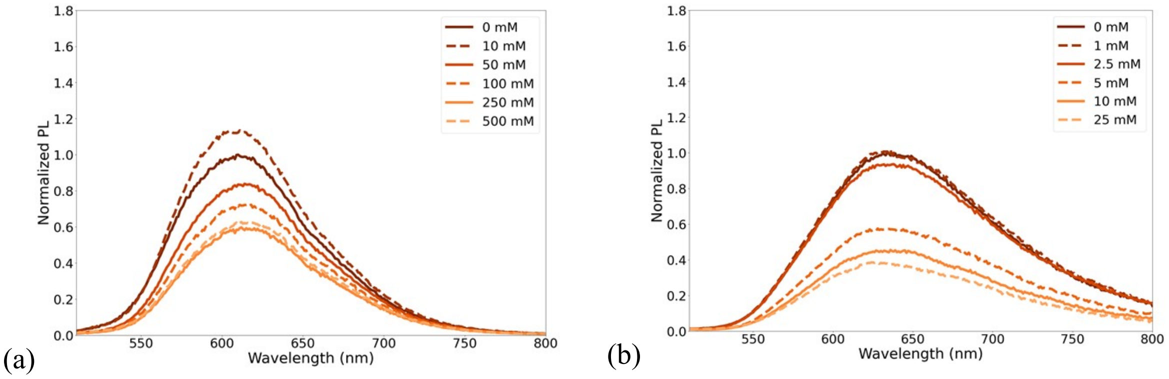


**Figure S17.** Fluorescence quenching spectra for as-synthesized POZ-M(+) NPs with (a) L-cysteine (10−500 mM) or (b) methyl viologen (1−25 mM) under λexc. =495 nm. Fluorescence quenching measurements allow us to distinguish between the deactivation of the excited state of organic NPs via electron transfer from an electron donor (L-cysteine, reductive quenching cycle) or via electron transfer to an electron acceptor (Methyl viologen, oxidative quenching cycle). The gradual addition of small quantities of methyl viologen resulted in obvious excited state quenching, while much higher quantities of L-cysteine were necessary to quench the excited state of POZ-M (+) NPs. This result suggests that POZ-M NPs should undergo oxidative quenching cycle, where at first excited POZ-M NPs reduces methyl viologen, and then oxidized POZ-M (+) NPs are regenerated by electron transfer from L-cysteine.

**Table S2.** The table comparing the performance of POZ-M(+) NRs with the related state-of the art systems.

| Photosensitizer | Catalyst | Sacrificial Electron Donor | Solvent | Light (mW/cm$^2$) | TON | Activity (μmol·g$^{-1}$·h$^{-1}$) | Stability | Ref. |
|---|---|---|---|---|---|---|---|---|
| **POZ-M(+) nanorods** | CODH (II) *1s$^{-1}$@pH8 *0.08 @pH 5.7 | Cysteine pH 5.7 | $H_2O$ | LED (400 – 750 nm) 50 mW/cm$^2$ | 8224 (with MV2+) 120000 (with DQ-OH) | **1140** | 150 h | This Study |
| **Organic photosensitizers used in $CO_2$ reduction to CO** | | | | | | | | |
| **PFBT Pdots** | - | 0.2M TEOA | $H_2O$ | LED (400 – 750 nm) 50 mW/cm$^2$ | 58 | **57** | 25 h | 4 |
| **Triphenylamine based conjugated microporous polymers** | - | $H_2O$ | Deposited as film | 300 W Xe lamp | - | **37** | 20 h | 5 |
| **Triazine based polymer** | - | TEOA | ACN to $H_2O$ = 4:1 | 300 W Xe lamp | - | **18** | ~20 h | 6 |
| **Pyrene-based polymer** | - | TEOA | Ionic liquid | 220 W Xe lamp | - | **47** | 50 h | 7 |
| **PNDI-BP** | - | $H_2O$/TEA (vol. 1/1) | $H_2O$ | 100 mW·cm$^{-2}$ | - | **5** | 330 h | 8 |
| **PSP** | PSP2 Nickel–terpyridine | BIH | $H_2O$ | Solar simulator (λ>400nm) | 120 | **-** | 12h | 9 |
| **CODH(I) and CODH(II) catalysts (* represents intrinsic enzyme activity)** | | | | | | | | |
| **RuP@TiO$_2$** | CODH (I) | MES | $H_2O$ | Kodak Carousel S-AV 1010 projector with a 420 nm long-pass filter, 45 mW/cm$^2$ | ~2000 | - | 4 h | 10 |
| **CdS-NRs** | CODH (I) | EDTA | $H_2O$ | Kodak Carousel S-AV 1010 projector with a 420 nm long-pass filter,23 mW/cm$^2$ | ~22000 | - | 5 h | 11 |
| **CdS-QDs** | CODH (I) | MES | $H_2O$ | Kodak Carousel S-AV 1010 projector with a 420 nm long-pass filter 23 mW/cm$^2$ | ~4500 | - | 5 h | 11 |
| **Ag NCs@TiO$_2$** | CODH (I) *95s$^{-1}$ at pH 6.0 | 0.1M TEOA, 0.1M NaCl, and 25 mM EDTA at pH 6.0. | $H_2O$ | A 300W arc lamp (Newport 67005)fitted with a 420 nm filter | ~250000 | - | 6 h | 12 |
| **CdSe/CdS Nanorod** | CODH (II) *567s$^{-1}$ at pH 7.5 | 3-mercapto-propionic acid | $H_2O$ | 445 nm diode laser | 7740 | - | 1 h | 13 |

**References:**


1 M. V Pavliuk, S. Wrede and H. Tian, *Chem. Commun.*, 2023, **59**, 5611–5614.

2 M. Benvenuti, M. Meneghello, C. Guendon, A. Jacq-Bailly, J.-H. Jeoung, H. Dobbek, C. Léger, V. Fourmond and S. Dementin, *Biochim. Biophys. Acta - Bioenerg.*, 2020, **1861**, 148188.

3 B. Xu, L. Tian, A. S. Etman, J. Sun and H. Tian, *Nano Energy*, 2019, **55**, 59–64.

4 B. Cai, M. Axelsson, S. Zhan, M. V Pavliuk, S. Wang, J. Li and H. Tian, *Angew. Chemie Int. Ed.*, 2023, **62**, e202312276.

5 C. Dai, L. Zhong, X. Gong, L. Zeng, C. Xue, S. Li and B. Liu, *Green Chem.*, 2019, **21**, 6606–6610.

6 C. Yang, W. Huang, L. C. da Silva, K. A. I. Zhang and X. Wang, *Chem. – A Eur. J.*, 2018, **24**, 17454–17458.

7 Y. Chen, G. Ji, S. Guo, B. Yu, Y. Zhao, Y. Wu, H. Zhang, Z. Liu, B. Han and Z. Liu, *Green Chem.*, 2017, **19**, 5777–5781.

8 S.-H. Wang, F. Khurshid, P.-Z. Chen, Y.-R. Lai, C.-W. Cai, P.-W. Chung, M. Hayashi, R.-J. Jeng, S.-P. Rwei and L. Wang, *Chem. Mater.*, 2022, **34**, 4955–4963.

9 X. Liu, F. Kang, C. Hu, L. Wang, Z. Xu, D. Zheng, W. Gong, Y. Lu, Y. Ma and J. Wang, *Nat. Chem.*, 2018, **10**, 1201–1206.

10 T. W. Woolerton, S. Sheard, E. Pierce, S. W. Ragsdale and F. A. Armstrong, *Energy Environ. Sci.*, 2011, **4**, 2393–2399.

11 Y. S. Chaudhary, T. W. Woolerton, C. S. Allen, J. H. Warner, E. Pierce, S. W. Ragsdale and F. A. Armstrong, *Chem. Commun.*, 2012, **48**, 58–60.

12 L. Zhang, M. Can, S. W. Ragsdale and F. A. Armstrong, *ACS Catal.*, 2018, **8**, 2789–2795.

13 D. W. White, D. Esckilsen, S. Kyu Lee, S. W. Ragsdale and R. Brian Dyer, *J. Phys. Chem. Lett.*, 2022, **13**, 5553–5556.
